# Carcass conformation and fat cover scores in beef cattle: A comparison of threshold linear models vs grouped data models

**DOI:** 10.1186/1297-9686-43-16

**Published:** 2011-05-14

**Authors:** Joaquim Tarrés, Marta Fina, Luis Varona, Jesús Piedrafita

**Affiliations:** 1 Grup de Recerca en Remugants, Departament de Ciència Animal i dels Aliments, Universitat Autònoma de Barcelona, 08193 Bellaterra, Spain; 2 Unidad de Genética Cuantitativa y Mejora Animal, Departamento de Anatomía, Embriología y Genética, Universidad de Zaragoza, 50013 Zaragoza, Spain

## Abstract

**Background:**

Beef carcass conformation and fat cover scores are measured by subjective grading performed by trained technicians. The discrete nature of these scores is taken into account in genetic evaluations using a threshold model, which assumes an underlying continuous distribution called liability that can be modelled by different methods.

**Methods:**

Five threshold models were compared in this study: three threshold linear models, one including slaughterhouse and sex effects, along with other systematic effects, with homogeneous thresholds and two extensions with heterogeneous thresholds that vary across slaughterhouses and across slaughterhouse and sex and a generalised linear model with reverse extreme value errors. For this last model, the underlying variable followed a Weibull distribution and was both a log-linear model and a grouped data model. The fifth model was an extension of grouped data models with score-dependent effects in order to allow for heterogeneous thresholds that vary across slaughterhouse and sex. Goodness-of-fit of these models was tested using the bootstrap methodology. Field data included 2,539 carcasses of the Bruna dels Pirineus beef cattle breed.

**Results:**

Differences in carcass conformation and fat cover scores among slaughterhouses could not be totally captured by a systematic slaughterhouse effect, as fitted in the threshold linear model with homogeneous thresholds, and different thresholds per slaughterhouse were estimated using a slaughterhouse-specific threshold model. This model fixed most of the deficiencies when stratification by slaughterhouse was done, but it still failed to correctly fit frequencies stratified by sex, especially for fat cover, as 5 of the 8 current percentages were not included within the bootstrap interval. This indicates that scoring varied with sex and a specific sex per slaughterhouse threshold linear model should be used in order to guarantee the goodness-of-fit of the genetic evaluation model. This was also observed in grouped data models that avoided fitting deficiencies when slaughterhouse and sex effects were score-dependent.

**Conclusions:**

Both threshold linear models and grouped data models can guarantee the goodness-of-fit of the genetic evaluation for carcass conformation and fat cover, but our results highlight the need for specific thresholds by sex and slaughterhouse in order to avoid fitting deficiencies.

## Background

Beef cattle production is becoming increasingly concerned with meat and carcass quality traits [1]. Currently, beef cattle genetic evaluations include mainly growth traits, but carcass traits are also economically important [2]. European beef producers are paid based on the weight of the animals at slaughter and on carcass conformation (CON) and fat cover (FAT) scores. All carcasses are classified at commercial slaughterhouses according to CON and FAT scores measured by subjective grading performed by trained technicians. These subjective records usually involve classification under a categorical and arbitrarily predefined scale, which may lead to strong departures from the Gaussian distribution. Theoretically, the discrete nature of performance traits is taken into account in genetic evaluations using a threshold linear model [3], which assumes an underlying continuous distribution called liability. This model includes thresholds that link the underlying distribution with the observed scale. However, in some cases, different technicians may use different intervals on the categorical scale, or a wider or narrower range of values for the subjective grading. Thus, the link between the observed scale and the liability scale could be specific to each technician. In 2006, Varona and Hernandez [4] proposed a specific ordered category threshold linear model for sensory data and concluded that each panelist used a different pattern of categorization. In 2009, Varona et al. [5] compared different threshold linear models using the deviance information criterion and showed that the most plausible model to analyse carcass traits was the slaughterhouse specific ordered category threshold linear model. This result was confirmed by the fact that the threshold estimates differed notably between slaughterhouses.

Liability may follow many distributions, such as the Gaussian distribution (probit model), the logistic distribution (logit model) or the reverse extreme value distribution. This latter distribution is a log-Weibull distribution and the resulting model can therefore be framed as a linear model for the logarithm of the liability The Weibull distribution (including the exponential distribution as a special case) is commonly used in survival analysis and it can be parameterised as either a proportional hazards model or a log-linear model. It is the only family of distributions that has this property [6]. Whereas a proportional hazards model assumes that the effect of a covariate is to multiply the hazard by some constant, a log-linear model assumes that the effect of a covariate is to multiply the underlying variable by some constant [6]. The results of fitting a Weibull model can therefore be interpreted in both frameworks.

Prentice and Gloeckler [7] presented the "grouped data model" for analysis of discrete data while maintaining the assumption of proportional hazards. Ducrocq [8] reparameterized and extended grouped data models to include random effects for animal breeding applications. Tarres et al. [9] showed that Ducrocq's formulae [8], drawn from the grouped data model for survival analysis (where the value of the underlying variable is necessarily larger than 0), can be applied to an underlying variable with negative values. They also highlighted the flexibility of the grouped data model for the analysis of discrete traits, such as calving ease of beef calves, in comparison to homoscedastic and heteroscedastic threshold linear models.

Given the diversity of models to analyse discrete variables such as CON and FAT scores, comparing these models requires specific tools to test goodness-of-fit with real data. Bootstrap approaches, introduced by Efron [10], have become routine methods to approximate the distribution of a parameter of interest, and have been applied to the animal breeding framework [11,12]. In 2006, Casellas et al. [13] proposed a parametric bootstrap procedure to test goodness-of-fit that provides a clear framework to compare predicted and actual distributions of variables of interest. Significant fitting deficiencies are revealed when the distribution of the actual data is not included within the bootstrap interval. This bootstrap approach could be a very useful tool to validate models by direct assessment of the ability of the model to fit the actual data.

The aim of this work was to perform a parametric bootstrap procedure to test the goodness-of-fit of three threshold linear models, a threshold log-linear Weibull model, and a grouped data model for the analysis of carcass conformation and fat cover in beef cattle. The three threshold linear models were a model with slaughterhouse and sex effects, along with other systematic effects, with homogeneous thresholds, and two extensions with heterogeneous thresholds that vary across slaughterhouses and across slaughterhouse and sex.

## Methods

### Data

Bruna dels Pirineus is a beef type breed selected from the old Brown Swiss (derived from the Canton Schwyz) with herds located in the Pyrenean mountain areas of Catalonia (Spain). From October/November to June, when most of the calving occurs, the animals remain in the valleys close to the villages and then the cows and calves are taken to the mountains to graze alpine pastures. After weaning, calves are fattened by *ad libitum *feeding with barley-corn concentrate meal and straw. Data were recorded between 2004 and 2009 in 12 slaughterhouses located in Catalonia (Spain), and included records from 2,539 beef carcasses from animals participating in the Yield Recording Scheme of the breed. Two traits were analysed in this study: the CON score, which describes the development of essential parts of the carcass profile according to the (S)EUROP scale (CEE no 2930/81, 1981), and the FAT score, which quantifies the amount of fat on the outside of the carcass and in the thoracic cavity. The categorical scale of CON was converted to a numeric scale from 2.00 (O) to 5.00 (E) because S and P scores were not observed. Similarly, FAT could have scores between 1 and 5, but scores over 4 were not observed. The percentages of each score in each slaughterhouse are presented in Tables 1 and 2. The data were completed with pedigree records provided by the Bruna dels Pirineus Breeders Association (FEBRUPI). Both FEBRUPI and slaughterhouse databases were merged according to the European animal identification code. The pedigree file contained 5,153 animals related to these calves, of which 332 were sires. Statistical analysis of these data was performed with different threshold models.

### Threshold Linear animal Model (TLM)

Each CON and FAT score was modelled as a discrete variable *Y *conditional to an unobservable underlying continuous variable *T*, referred to as liability. The probability that the discrete variable *Y *has a value *k *is:

where *τ*_1_, *τ*_2 _and *τ*_3 _are thresholds that define the four categories of response. The prior distributions of the threshold positions were assumed to be flat. Thresholds *τ*_2 _and *τ*_3 _are assumed to be known, i.e. arbitrarily fixed to 0 and 2.0 for CON and FAT, to provide a simpler sampling scheme than the one defined by fixing the mean and the residual variance of the liability [14]. The posterior conditional distributions for the augmented underlying variables are censored normal distributions, as described by Sorensen et al. [15].

The underlying variable *T *had the following distribution:

where **β **are the regression coefficients of the systematic effects, **h **are herd effects, **u **direct breeding values, **X**, **Z_1_**, and **Z_2 _**are incidence matrices linking data with their respective effects, and  is the residual variance. The systematic effects included in **β**, i.e. , referred to slaughterhouse (12 levels), sex (males and females), parity (1st to 4^th ^or more), age at slaughter (6 levels: 9 to 14 months), season at slaughter (winter, spring, summer and autumn) and year of slaughter (2005 to 2009). Prior distribution for herd effects (73 levels) was assumed to be multivariate normal

where  is the herd variance. For direct breeding values, the prior distribution was:

where **A **is the numerator relationship matrix and  is the additive genetic variance. The prior distributions for systematic effects and the (co)variance components were bounded flat uniform distributions.

Bayesian analysis of the Threshold Linear Model (TLM) was carried out with the Gibbs sampler algorithm implemented in Varona et al. [5]. Each analysis consisted of a single chain of 100,000 iterations, with the first 25,000 samples discarded. Analysis of convergence and calculation of effective sample size followed the algorithms by Raftery and Lewis [16]. All iterations in the analysis were used to compute posterior means and standard deviations of estimated regression coefficients and random effects, so that all available information from the output of the Gibbs sampler could be considered.

### Specific Slaughterhouse Threshold Linear animal Model (SHTLM)

This model is the same as above, except that it estimates a specific set of thresholds for each slaughterhouse. Now, the probability that the discrete variable *Y *takes a value *k *is:

where *τ*_*sh*,1_, *τ*_*sh*,2 _and *τ*_*sh*,3 _are thresholds that define the four categories of response and have a different position depending on the slaughterhouse (12 different slaughterhouses). As in the previous model, the prior distributions of the threshold positions are assumed to be flat, and thresholds *τ*_12,2 _and *τ*_12,3 _are assumed to be known and arbitrarily fixed to 0 and 2.0 for both traits. The presence of specific thresholds for each slaughterhouse should take into account the variation captured by the slaughterhouse effect in TLM. Thus, in this model, systematic effects were reduced to sex, parity, age at slaughter, season and year at slaughter. Once again, a Bayesian analysis was carried out with the Gibbs sampler algorithm implemented as in Varona et al. [5].

### Specific Sex per Slaughterhouse Threshold Linear animal Model (SEXTLM)

This model differs from the previous ones in that it estimates a specific set of thresholds for each sex in each slaughterhouse. Now, the probability that the discrete variable *Y *takes a value *k *is:

where *τ*_*sex,sh*,1_, *τ*_*sex,sh*,2 _and *τ*_*sex,sh*,3 _are thresholds that define the four categories of response and have a different position depending on the interaction of sex and slaughterhouse (24 levels). As in the previous model, the prior distributions of the threshold positions are assumed to be flat, and thresholds *τ*_*male*,12,2 _and *τ*_*male*,12,3 _are assumed to be known and fixed to 0 and 2.0 for both traits. The presence of specific thresholds for each sex in each slaughterhouse should take into account the variation captured by the sex effect in SHTLM. Thus, in this model, systematic effects were reduced to parity, age at slaughter, season and year at slaughter. Once again, a Bayesian analysis was carried out with the Gibbs sampler algorithm implemented in Varona et al. [5].

### Threshold log Linear Weibull Model (TlogLWM)

In the previous models, CON and FAT scores were modelled as a discrete variable *Y *conditional to an unobservable underlying continuous variable *T*, referred to as liability that follows a linear model. In the TlogLWM, we assume that the liability is modelled as follows:

where *t*_0 _follows a standard Weibull distribution. In this case, this model is equivalent to:

where e **follows an extreme value distribution [17], and *ρ *and *λ *are the Weibull parameters, **β **are the regression coefficients of the systematic effects, **h **are herd effects, **u **are breeding values, and **X**, **Z_1_**, and **Z_2 _**are incidence matrices linking data with their respective effects. The systematic effects included in **β**, i.e. , were the same as in TLM. Here it is important to note the minus sign in front of the effects because it influences the interpretation of the results.

The probability that the discrete variable *Y *has a value *k *is:

where *τ*_1_, *τ*_2 _and *τ*_3 _are homogeneous thresholds that define the four categories of response and , with *h*(.) being the underlying hazard function that is the ratio of the probability density function to the complementary cumulative distribution function [8]. This hazard function follows a proportional hazard model *h*(*t*) = *h*_0_(*t*)exp(**Xβ**+**Z**_**1**_**h **+ **Z**_**2**_**u**) with *h*_0_(.) being the baseline Weibull hazard function.

In our data, each CON and FAT score can take four values k = 1, 2, 3 or 4. Then, the probability that the discrete variable Y has a value k was calculated as:

Because *α_k _*can by definition only take values between 0 and 1, it was modelled using a log-log transformation as:

where *μ_1_*, *μ_2 _*and *μ_3 _*were mean values ranging from -∞ to +∞. These means were different for each k value of CON and FAT while systematic effects **β**, herd effects **h **and breeding values **u **were the same for all the k values

The Survival Kit package [18] was used to analyse the TlogLWM model because the likelihood expression was exactly the same as assuming an underlying variable T with a threshold proportional hazard model [8]. In fact, TlogLWM is a particular case of a threshold proportional hazard model with a baseline Weibull distribution.

### Grouped Data Model (GDM)

The threshold proportional hazard models are called grouped data models [8]. In these models, the discrete variables *Y *are modelled conditional to an unobservable liability that follows a proportional hazard model. In this case, the hazard function of the liability *h*(*t*) = *h*_0_(*t*)exp(**Xβ **+ **Z**_**1**_**h **+ **Z**_**2**_**u**) is the product of two terms, the baseline hazard function *h*_0_(.) and the regression coefficients term. Unlike in the previous model, in GDM the baseline distribution of the underlying variable *T *can be unknown and not necessarily Weibull, because the estimates of regression coefficients, herd and genetic effects will be exactly the same regardless of the distribution assumed.

The probability that the discrete variable Y has a value k was calculated as before:

where *τ*_*sex,sh*,1_, *τ*_*sex,sh*,2 _and *τ*_*sex,sh*,3 _are heterogeneous thresholds that vary by slaughterhouse and sex and define the four categories of response, and *α_k _*was modelled using a log-log transformation as:

where *μ*_1_, *μ*_2 _and *μ*_3 _were mean values ranging from -∞ to +∞. In our study, the variables included in **β **were the systematic effects with incidence matrix **X**, i.e. . On the one hand, these regression coefficients were the same for all values k of CON and FAT. On the other hand, the slaughterhouse and sex effects were assumed to be score-dependent, i.e. different for each value *k *of CON and FAT scores. Likelihood ratio tests determined whether including score-dependent effects for these factors gave a significantly better fit. Herd effects **h **and breeding values **u **were assumed to be random with incidence matrices **Z_1 _**and **Z_2 _**that link data with their respective effects. Prior distributions for herd effects and genetic effects were chosen as in the previous models. The Survival Kit package [18] was used for the analysis of the GDM model.

It is important to note here that the heterogeneous threshold positions do not appear in the likelihood expression and therefore they are not estimated. However, they can be calculated a posteriori by assuming a known distribution and solving ln *α_k _*= ln *S*(*τ_sex,sh,k_*) - ln *S*(*τ_sex,sh,k-1_*) where *S*(.) is the complementary cumulative distribution function of the liability. In this way, a direct relationship can be established between score-dependent effects and heterogeneous thresholds positions.

### Parametric bootstrapping for model comparison

A parametric bootstrap approach was applied to test the goodness-of-fit of the described models in the analysis of CON and FAT scores. The bootstrapping methodology was the same as in Tarres et al. [9]. Confidence intervals obtained for the frequency of each *k *value of CON and FAT were stated as being the 0.025 and 0.975 percentiles of the bootstrap samples, and they were easily contrasted with the frequencies of the actual data. Significant fitting deficiencies were revealed when the actual frequencies were outside the confidence interval for one model, and they could be statistically quantified through the bootstrapped p-values [19].

## Results

### Descriptive statistics

The average carcass of the Bruna dels Pirineus breed under commercial conditions weighed around 279 kg at 12.5 months of age (377 d), with an average CON score of 3.43, between R (good) and U (very good), and a low FAT average score (2.48). Male calves were slaughtered one month later than females (387 d vs. 360 d) and had a higher cold carcass weight (305 kg vs. 231 kg) and CON score (3.61 vs 3.35) but a slightly lower FAT average (2.47 vs 2.54) (results not shown in tables). These results show that under commercial conditions the Bruna dels Pirineus and the Pirenaica breeds have similar performances [20], which are also similar to those previously reported for the same breeds under an experimental environment by Piedrafita et al. [21]. In addition, the Bruna dels Pirineus breed results were comparable to those from other European populations scored by the EUROP carcass classification system, such as the Swedish Charolais and Simmental populations studied by Eriksson et al. [1], but with a higher CON score and a smaller FAT score than the Irish populations studied by Hickey et al. [2].

### Threshold Linear animal Model (TLM)

A standard alternative for analysis of categorical data such as CON and FAT scores is the threshold linear model or TLM [3-5]. Using TLM, sex, parity and age at slaughter effects reflected the expected physiological relationship among them (results not shown). Males showed larger CON scores than females, which is very similar to results of Altarriba et al. [20]. The situation was reversed for FAT, since females showed a higher FAT score than males, due to their greater precocity [22]. Calves from multiparous dams had higher CON scores than calves from primiparous dams, but these differences were not so large for FAT scores. Moreover, for the effect of age at slaughter, an almost linear increasing relationship was observed for CON scores (results not shown) but for FAT scores no clear tendency was detected. The difference in precocity among sexes did not generate a different effect of age at slaughter on FAT score between sexes because this interaction was not significant in our data. Finally, significant differences in CON and FAT scores were detected depending on the season and year of slaughter but there was no clear trend over time.

These estimated regression coefficients were used to compute the bootstrap intervals for TLM. Significant fitting deficiencies were revealed because in many cases the actual frequency of CON and FAT scores was not within the bootstrap interval, especially when stratifying by slaughterhouse (Tables 1 and 2). This was because CON and FAT score frequencies varied significantly between slaughterhouses. For two slaughterhouses (11 and 12), over 80% of the carcasses were qualified as R for CON, whereas in the other slaughterhouses most of the carcasses were qualified as U (Table 1). In the case of FAT scores, several slaughterhouses (1, 3, 4, 5, 8, 9 and 12) qualified most carcasses with a value of 3, while in some slaughterhouses (2, 6, 7 and 10) the most frequent value was 2, and in one slaughterhouse (11) the most frequent value was 1 (Table 2). These differences among slaughterhouses can be explained either by the fact that some slaughterhouses prefer to slaughter light young animals (i.e less than one year old) compared to other slaughterhouses, or by the fact that both traits were scored by different technicians in each slaughterhouse. Despite the existence of an objective European scoring system, each technician may have a different subjective interpretation (i.e. each technician puts the threshold at a different position). As in Varona et al. [5], this fact reveals the complexity of the normalization of carcass evaluation for CON and FAT scores, which cannot be accommodated by the TLM because it suffers from low flexibility due to the assumptions made in the model (i.e. all the slaughterhouses have the same threshold position).

**Table 1:** Percentages of carcass conformation stratified by slaughterhouse

Slaughterhouse	Carcass conformation			
	
	O	R	U	E
1	1.10	34.62	64.29	0.00
	(0.00-0.01) ***	(35.85-48.63) **	(47.80-60.99) **	(0.82-6.04) ***
2	1.90	36.08	47.47	14.56
	(0.00-0.32) ***	(28.80-41.46)	(50.63-65.19) **	(3.80-11.08) **
3	1.25	38.13	59.38	1.25
	(0.00-0.31) ***	(38.75-48.59) *	(48.91-59.06) *	(0.78-4.06)
4	0.60	33.13	56.63	9.64
	(0.00-0.00) **	(25.90-38.86)	(54.22-68.07)	(3.01-10.24)
5	0.00	50.53	49.47	0.00
	(0.00-0.00)	(43.16-62.63)	(36.32-55.79)	(0.00-3.68)
6	0.00	7.75	76.76	15.49
	(0.00-0.00)	(4.93-14.44)	(65.85-80.63)	(11.62-23.59)
7	0.00	36.90	59.52	3.57
	(0.00-0.00)	(26.19-46.43)	(50.60-70.83)	(0.00-6.55)
8	0.00	17.65	69.41	12.94
	(0.00-0.00)	(10.59-25.29)	(59.71-78.82)	(6.47-20.00)
9	1.82	7.27	40.00	50.91
	(0.00-0.00) ***	(0.00-8.18)	(43.64-68.18) **	(29.09-52.73)
10	0.00	50.00	50.00	0.00
	(0.00-0.00)	(35.42-70.83)	(27.08-64.58)	(0.00-6.25)
11	0.00	96.36	2.96	0.68
	(0.34-2.28) *	(92.03-96.24) *	(2.73-6.49)	(0.00-0.11) ***
12	0.25	80.99	18.00	0.76
	(0.00-0.63)	(80.61-85.55)	(14.13-18.95)	(0.00-0.32) **

Overall	0.51	58.29	36.63	4.57
	(0.10-0.51)	(58.37-61.22) *	(34.54-37.64)	(3.17-4.51) *

Bootstrap confidence intervals (95%) in parentheses, and p-values from a threshold linear model (TLM). Percentage outside the bootstrap interval if * (P < 0.05); ** (P < 0.01); *** (P < 0.001)

**Table 2:** Percentages of fat cover stratified by slaughterhouse

Slaughterhouse	Fat cover			
	
	1	2	3	4
1	0.00	0.00	100.00	0.00
	(0.00-0.00)	(0.30-5.49) *	(91.16-97.87) **	(0.30-5.18) *
2	6.04	55.03	38.93	0.00
	(2.35-9.40)	(46.98-62.42)	(32.55-46.81)	(0.00-0.00)
3	1.26	24.84	73.90	0.00
	(0.00-1.42)	(19.34-28.30)	(70.91-80.03)	(0.00-0.79)
4	0.00	0.00	100.00	0.00
	(0.00-0.00)	(0.31-5.59) *	(90.68-97.83) **	(0.31-5.59) *
5	0.00	4.21	95.79	0.00
	(0.00-0.53)	(1.58-9.47)	(88.42-98.42)	(0.00-4.74)
6	0.99	87.13	11.88	0.00
	(4.95-16.83) ***	(58.91-77.72) ***	(13.86-29.21) **	(0.00-0.00)
7	5.00	65.00	30.00	0.00
	(0.83-12.50)	(50.83-75.00)	(20.00-43.33)	(0.00-0.00)
8	0.00	38.27	60.49	1.23
	(0.00-3.09)	(22.84-42.59)	(56.79-76.54)	(0.00-1.23)
9	0.00	5.45	90.91	3.64
	(0.00-0.00)	(0.00-8.18)	(88.18-99.09)	(0.00-6.36)
10	0.00	95.83	4.17	0.00
	(2.08-27.08) *	(50.00-85.42) **	(4.17-33.33)	(0.00-0.00)
11	69.57	25.32	4.60	0.51
	(62.40-70.97)	(27.62-36.45) **	(0.38-2.56) ***	(0.00-0.00) ***
12	0.53	12.89	79.21	7.37
	(0.00-0.39) **	(5.26-10.53) **	(86.32-92.37) ***	(1.32-4.34) ***

Overall	14.70	25.11	58.51	1.67
	(13.62-15.59)	(22.51-25.64)	(58.77-61.55) *	(0.68-1.59) *

Bootstrap confidence intervals (95%) in parentheses, and p-values from a threshold linear model (TLM). Percentage outside the bootstrap interval if * (P < 0.05); ** (P < 0.01); *** (P < 0.001)

### Specific Slaughterhouse Threshold Linear animal Model (SHTLM)

The flexibility of threshold models was improved in SHTLM by estimating different thresholds per slaughterhouse in order to take the different subjective interpretations of scoring systems into account. The posterior means for the thresholds indicated a large variation among slaughterhouses (results not shown), in strong concordance with the heterogeneity of the raw data presented in Tables 1 and 2. Threshold position *τ*_*sh*,3 _was negative for slaughterhouses in which most carcasses were qualified as U for CON and positive for slaughterhouses in which most carcasses were qualified as R. For FAT, the threshold position *τ*_*sh*,1 _was positive for slaughterhouse 11, in which most carcasses were qualified as 1 (69.57%), and the threshold position *τ*_*sh*,2 _was over 0.45 for slaughterhouses (2, 6, 7 and 10) in which most carcasses were qualified as 2. Using SHTLM, most of the fitting deficiencies when stratifying by slaughterhouse disappeared, as most of the frequencies of CON and FAT scores from actual data fell within the bootstrap intervals (results not shown). However, SHTLM still failed to correctly fit the frequencies by sex (Tables 3 and 4), especially for FAT score, since five of the eight actual percentages in Table 4 were not within the bootstrap interval. This fact indicates that the threshold positions for FAT scores differed by sex and that differences among sexes could not be totally captured by a systematic effect, as fitted in SHTLM.

**Table 3:** Percentages of carcass conformation stratified by sex

SEX	Carcass conformation			
	
	O	R	U	E
Males	0.25	49.88	43.89	5.99
TLM	(0.00-0.28)	(49.53-53.21)	(40.99-45.07)	(4.52-6.48)
SHTLM	(0.00-0.22) *	(49.45-52.88)	(41.29-45.04)	(4.64-6.58)
SEXTLM	(0.00-0.28)	(49.34-52.81)	(41.08-44.76)	(4.89-6.92)
TlogLWM	(0.00-0.64)	(49.50-53.26)	(41.12-45.17)	(4.40-6.37)
GDM	(0.03-0.56)	(49.47-53.30)	(41.24-45.29)	(4.18-6.02)
Females	0.96	72.73	24.17	2.14
TLM	(0.16-1.18)	(72.03-76.52)	(21.87-26.47)	(0.43-1.63) **
SHTLM	(0.11-0.96)	(71.39-75.78)	(22.78-27.11)	(0.43-1.60) **
SEXTLM	(0.16-0.96)	(72.09-76.41)	(21.55-25.94)	(0.91-2.14)
TlogLWM	(0.18-1.11)	(72.05-76.53)	(22.02-27.47)	(0.45-1.62) **
GDM	(0.37-1.60)	(71.18-75.67)	(21.76-26.26)	(0.86-2.38)

Bootstrap confidence intervals (95%) in parentheses, and p-values from a threshold linear model (TLM), a specific slaughterhouse threshold linear model (SHTLM), a specific sex per slaughterhouse threshold linear model (SEXTLM), a threshold log linear Weibull model (TlogLWM), and a grouped data model (GDM)

Percentage outside the bootstrap interval if * (P < 0.05); ** (P < 0.01); *** (P < 0.001)

**Table 4:** Percentages of fat cover values stratified by sex

SEX	FAT			
	
	1	2	3	4
Males	11.80	29.79	57.92	0.50
TLM	(12.42-14.73) **	(23.31-27.52) ***	(58.58-62.29) **	(0.21-0.99)
SHTLM	(12.05-14.03) **	(25.74-29.70) *	(56.60-60.27)	(0.37-1.36)
SEXTLM	(10.48-12.62)	(28.30-32.30)	(55.52-59.20)	(0.33-1.28)
TlogLWM	(12.21-14.56) **	(23.56-27.79) ***	(58.34-62.01) **	(0.23-1.00)
GDM	(11.01-13.16)	(28.03-32.10)	(55.65-59.24)	(0.12-0.74)
Females	19.30	17.73	59.45	3.52
TLM	(14.41-18.06) **	(19.56-24.45) **	(57.69-61.77)	(1.11-3.06) **
SHTLM	(15.58-19.04) **	(18.25-23.08) **	(57.56-61.86)	(1.43-3.32) **
SEXTLM	(18.19-21.51)	(14.66-19.04)	(58.47-62.38)	(1.89-3.98)
TlogLWM	(14.93-18.52) **	(18.99-23.67) **	(57.55-61.67)	(1.22-3.15) **
GDM	(18.25-21.84)	(16.17-20.93)	(56.45-60.82)	(1.83-3.85)

Bootstrap confidence intervals (95%) in parentheses, and p-values from a threshold linear model (TLM), a specific slaughterhouse threshold linear model (SHTLM), a specific sex per slaughterhouse threshold linear model (SEXTLM), a threshold log linear Weibull model (TlogLWM), and a grouped data model (GDM)

Percentage outside the bootstrap interval if * (P < 0.05); ** (P < 0.01); *** (P < 0.001)

### Specific Sex per Slaughterhouse Threshold Linear animal Model (SEXTLM)

The flexibility of threshold models was improved in SEXTLM by estimating different thresholds per sex in each slaughterhouse in order to take the different subjective interpretations of scoring systems by sex into account. Using SEXTLM, the frequencies of CON and FAT scores by sex were always within the boostrapped boundaries (Tables 3 and 4) and no fitting deficiencies were detected. This fact confirmed that the interpretation of the scoring system was different for each sex in each slaughterhouse.

### Threshold log Linear Weibull Model (TlogLWM)

This model assumed proportional (log-linear) effects on CON and FAT scores, instead of the additive effects assumed in the threshold linear models, but again slaughterhouse, sex, parity, age at slaughter, season and year had a significant effect on CON and FAT scores. Male calves had a CON score 1.08 times higher than females, but females had a FAT score 1.03 times higher than males. Calves from multiparous dams had a CON score 1.08 times higher than calves from primiparous dams, and calves slaughtered over 14 months of age had a CON score 1.16 times higher than calves slaughtered before 9 months of age. In spite of the fact that these effects reflect the expected physiological relationship with CON and FAT scores, in the bootstrap analysis, TlogLWM failed to correctly fit the frequencies when stratifying by slaughterhouse and sex, especially for FAT (Tables 1 and 2). This fact again indicates that differences in CON and FAT scores among slaughterhouses and sexes could not be totally captured by a systematic effect, as fitted in TlogLWM, and heterogeneous thresholds should be allowed for sex and slaughterhouse effects.

### Grouped Data Model (GDM)

The previous model TlogLWM is a particular case of a grouped data model with a baseline Weibull distribution. Its fitting deficiencies can be solved in GDM by assuming that slaughterhouse and sex effects are score-dependent. Likelihood ratio tests confirmed this fact and showed that slaughterhouse and sex effects were significantly score-dependent, especially for FAT score (P < 0.001). Again, this fact reveals the complexity of normalising carcass evaluations for CON and FAT among slaughterhouses and sexes. In the bootstrap analysis, fitting deficiencies were not observed using GDM, as the frequencies of both traits when stratifying by each factor were always within the bootstrapped boundaries (Tables 3 and 4 for sex, and results not shown for the other factors). Including score-dependent effects gave great flexibility to GDM [9], and is similar to assume different thresholds positions by slaughterhouse and sex in threshold linear models, i.e. estimating one parameter for each score. Thus, this is a useful way to improve the goodness-of-fit of the models with a small increase in the number of parameters to be estimated, since there were only four scores.

### Heritabilities and EBV correlations among models

Estimates of variance components for the two traits are presented in Table 5. In this study, only slight differences in terms of variance components were noted among models (except for σ_h_^2^). Estimated heritabilities were similar for all models and ranged from 0.29 (SEXTLM) to 0.35 (TlogLWM) for the CON score, and from 0.21 (SHTLM) to 0.25 (TLM) for the FAT score (Table 5). These heritabilities estimates indicate that a sizeable fraction of the variance is additive genetic and confirmed that the results obtained were within the range of estimates from previous studies for the same subjective traits in other populations evaluated with the EUROP system [1,2,5,20].

**Table 5:** Heritability estimates for carcass conformation and fat cover

		TLM	SHTLM	SEXTLM	TlogLWM	GDM
CON	σ_u_^2^	0.344	1.206	1.668	0.621	0.609
	σ_h_^2^	0.089	0.548	0.735	0.180	0.180
	σ_e_^2^	0.666	2.304	3.238	1	1
	h^2^	0.313	0.300	0.291	0.345	0.340

FAT	σ_u_^2^	0.092	0.131	0.144	0.306	0.306
	σ_h_^2^	0.037	0.063	0.088	0.151	0.170
	σ_e_^2^	0.245	0.451	0.454	1	1
	h^2^	0.245	0.205	0.207	0.210	0.207

Estimated additive (σ_u_^2^), herd (σ_h_^2^) and error (σ_e_^2^) variances and heritabilities (h^2^) for carcass conformation (CON) and fat cover (FAT) under a threshold linear model (TLM), a specific slaughterhouse threshold linear model (SHTLM), a specific sex per slaughterhouse threshold linear model (SEXTLM), a threshold log linear Weibull model (TlogLWM), and a grouped data model (GDM)

**Figure 1:**
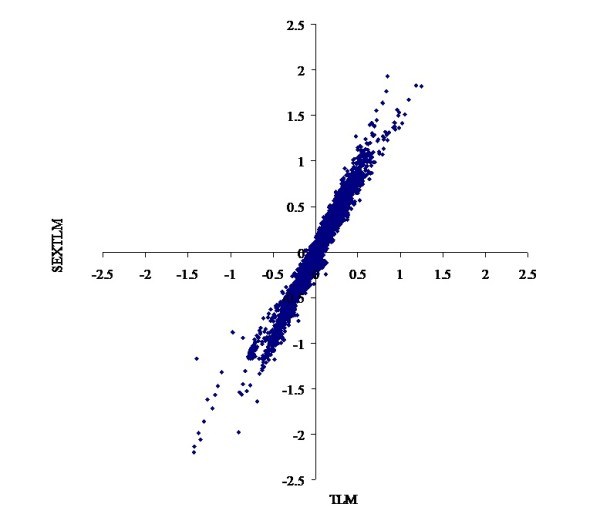
**Bivariate plot of estimated breeding values for carcass conformation**. Comparison of the threshold linear model and the specific sex by slaughterhouse threshold linear model

**Figure 2:**
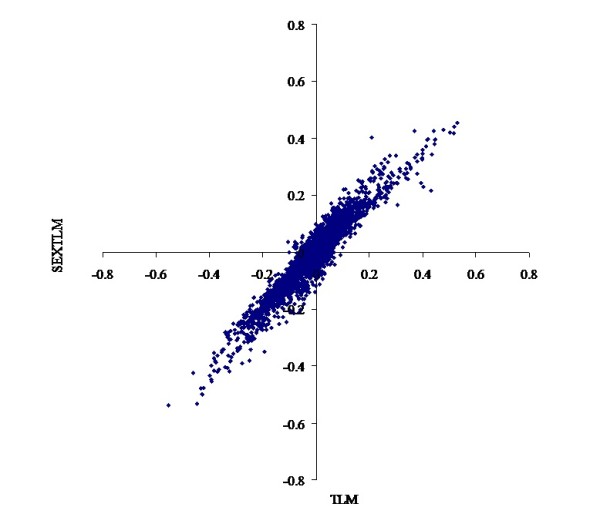
**Bivariate plot of estimated breeding values for fat cover**. Comparison of the threshold linear model and the specific sex by slaughterhouse threshold linear model

**Figure 3:**
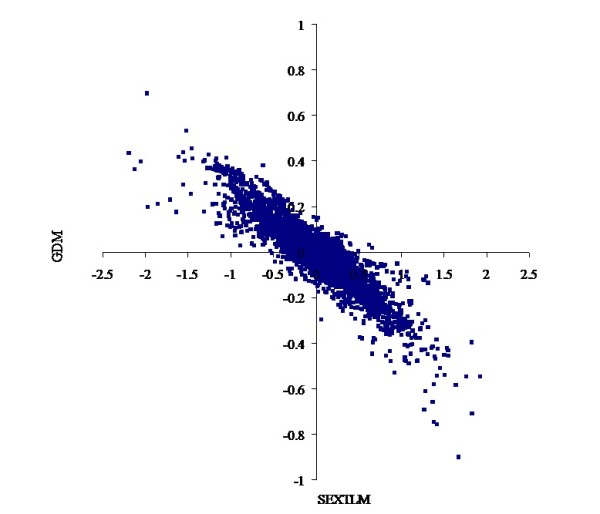
**Bivariate plot of estimated breeding values for carcass conformation**. Comparison of the specific sex by slaughterhouse threshold linear model and the grouped data model

**Figure 4:**
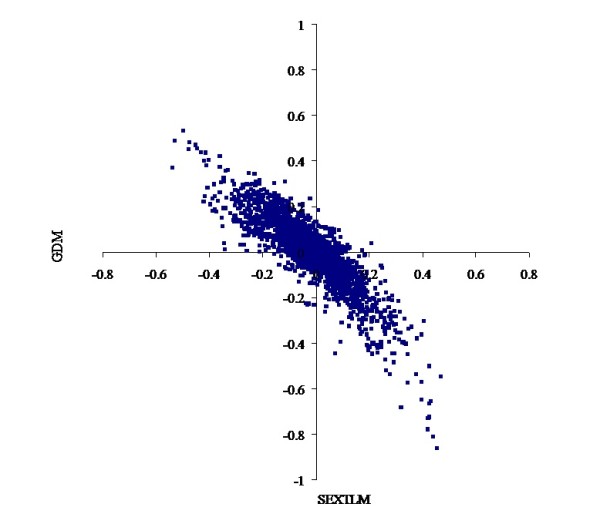
**Bivariate plot of estimated breeding values for fat cover**. Comparison of the specific sex by slaughterhouse threshold linear model and the grouped data model

The heterogeneity of the models described above had a marked impact on the prediction of EBV. For threshold linear models, the correlations were over 0.98 for CON and 0.95 for FAT scores between EBV from TLM and SEXTLM (Figures 1 and 2), much higher than the results of Varona et al. [5]. For grouped data models, the correlations were over 0.98 for CON and 0.96 for FAT scores between EBV from TlogLWM and GDM. Correlations between EBV from SEXTLM and GDM dropped to around minus 0.90 (Figures 3 and 4) because the assumptions made in both models were different. Whereas SEXTLM assumes that the effect of the EBV is additive on the underlying variable, a GDM assumes that the effect of the EBV is exponentiated to multiply the underlying variable by some constant. The correlations between EBV from SEXTLM and GDM were negative because a negative EBV for an animal in GDM meant higher CON and FAT scores, e.g. an EBV of -0.20 meant exp(-(-0.20)) = 1.22 times higher performance. However, although the prediction of EBV was different, both models can be used to analyse CON and FAT scores with a correct goodness-of-fit. Therefore, there is a need for an appropriate procedure, e.g. predictive ability criteria, to rank models properly for a better choice of the model for genetic evaluation.

## Conclusions

Significant fitting deficiencies were revealed when analyzing carcass conformation and fat cover scores using a threshold linear model with homogeneous thresholds. When a specific sex by slaughterhouse threshold model was considered, the fitting deficiencies were solved. Similar results were also obtained when heterogeneous thresholds were assumed in grouped data models that estimate score-dependent sex and slaughterhouse effects. The estimated heritabilities obtained from all models indicated that a sizeable fraction of the variance of both traits was additive genetic. Besides a goodness-of-fit procedure such as the one used in this work, an appropriate procedure, e.g. predictive ability criteria, to rank models properly for genetic evaluation in large field applications is needed.

## List of abbreviations used

CON: carcass conformation; EBV: estimated breeding values; FAT: fat cover; GDM: grouped data model; SEXTLM: specific sex per slaughterhouse threshold linear model; SHTLM: specific slaughterhouse threshold linear model; TLM: threshold linear model; TlogLWM: threshold log-linear Weibull model.

## Competing interests

The authors declare that they have no competing interests.

## Authors' contributions

JT performed the statistical analysis and drafted the manuscript. MF managed the YRS of the Bruna dels Pirineus breed and revised the manuscript critically for intellectual content. LV implemented software for the analysis of threshold traits and revised the manuscript critically for intellectual content. JP supervised the YRS, promoted the study and revised the manuscript critically for intellectual content. All authors read and approved the final manuscript.
